# Exploratory Analysis of Factors Affecting 30-Day, 90-Day, and 1-Year Readmission After Surgical Treatment of Primary Spinal Infection in Adults

**DOI:** 10.3390/jcm15041600

**Published:** 2026-02-19

**Authors:** Ismail Ertan Sevin, Selin Bozdag, Onur Davut Dag, Ibrahim Eralp Sevin, Pelin Pugar, Tuna Demirdal, Hasan Kamil Sucu

**Affiliations:** 1 Department of Neurosurgery, Ataturk Training and Research Hospital, Izmir Katip Çelebi University, 35360 Karabağlar, Izmir, Turkey; seviner67@yahoo.com (I.E.S.); selin.bzdg@gmail.com (S.B.); onurdavut.dag@saglik.gov.tr (O.D.D.); eralpsevin@gmail.com (I.E.S.); pelinpugar98@gmail.com (P.P.); 2 Department of Infectious Diseases, Ataturk Training and Research Hospital, Izmir Katip Çelebi University, 35360 Karabağlar, Izmir, Turkey

**Keywords:** primary spinal infection, spine surgery, readmission

## Abstract

**Background***:* Unplanned hospital readmission after surgical treatment of primary spinal infections (PSIs) represents a major clinical and economic burden. Despite the fact that advances in surgical and antimicrobial management have been made, risk stratification for early and late readmissions remains poorly defined. **Objective**: We aimed to explore the clinical, microbiological, and perioperative characteristics potentially associated with 30-day, 90-day, and 1-year unplanned readmissions following the surgical treatment of PSIs in adult patients. **Methods**: A retrospective cohort study was performed that included adult patients who underwent surgery for primary spinal infections between January 2017 and December 2023 at our tertiary referral center. Demographics, comorbidities, laboratory parameters, microbiological profiles, and postoperative outcomes were analyzed. Associations between candidate variables and readmission were explored using univariate statistical analyses; multivariable modeling was not performed due to the low number of readmission events. **Results**: In total, seventy-nine patients (mean age 62.2 ± 12.7 years; 38% female) were included. The in-hospital mortality rate was 5.1%; at 1-year follow-up, 10.3% of patients were readmitted and 5.9% required reoperation; and *Staphylococcus aureus* was the most common isolated pathogen. No independent variables demonstrated a statistically significant association with readmission. However, trends toward higher readmission were observed in patients with liver disease, hypoalbuminemia, and postoperative transfusion. **Conclusions**: In this exploratory single-center cohort, the low number of readmission events limited statistical power and precluded adjusted modeling. Univariate analyses did not identify statistically significant associations between the evaluated variables and 30-day, 90-day, or 1-year readmission; therefore, the results should be interpreted cautiously as hypothesis-generating. Larger prospective multicenter studies with adequate event counts are needed to support adjusted risk stratification approaches. Until such tools are available, close postoperative follow-up across all PSI patients is necessary.

## 1. Introduction

Despite the advances that have been made in diagnostic imaging, surgical techniques, and antimicrobial therapy, primary spinal infections (PSIs) remain a serious and potentially life-threatening condition. These infections encompass a heterogeneous group of entities including vertebral osteomyelitis, discitis, spondylodiscitis, epidural and intradural abscesses, and paraspinal soft-tissue infections [1]. Although the reported incidence remains relatively low, a consistent increase has been observed in recent decades, largely attributed to aging populations, immunosuppression, and the expanding burden of chronic systemic diseases [2].

Patients with PSIs often present with complex medical comorbidities such as diabetes mellitus, chronic renal failure, cardiovascular disease, malignancy, and immunosuppression. These factors not only predispose individuals to infection but also adversely affect postoperative recovery and long-term outcomes. Microbiological diversity further complicates management, with pathogens ranging from common bacteria such as Staphylococcus aureus to mycobacteria and opportunistic fungi, particularly in immunocompromised hosts [3,4,5,6].

Hospital readmission after spinal infection surgery represents a critical quality metric reflecting postoperative complications, persistent infection, implant failure, and systemic vulnerability. Early unplanned readmissions (within 30–90 days) are of particular importance because they frequently indicate early treatment failure or potentially preventable postoperative complications and contribute substantially to patient morbidity and healthcare utilization. Late readmissions often reflect mechanical instability or delayed treatment failure. Despite the clinical relevance, predictors of readmission following surgical treatment of PSIs remain insufficiently defined, and the available evidence is largely derived from heterogeneous patient cohorts and multi-indication spine surgery series [7,8,9,10].

Given the fact that only a limited number of disease-specific data are available, particularly in surgically treated PSIs, further investigation is warranted to better characterize readmissions and the factors potentially associated with them. Therefore, the aim of this exploratory study was to describe 30-day, 90-day, and 1-year unplanned readmission rates and to explore potential associations between demographic, clinical, laboratory, perioperative, and microbiological variables and readmission following the surgical treatment of primary spinal infections in an adult population.

## 2. Materials and Methods

This study was designed as a retrospective observational cohort analysis to describe unplanned hospital readmissions after surgical treatment of PSIs and explore potential associations between patient- and treatment-related variables and readmission. All patients who underwent surgery for PSI between January 2017 and December 2023 at Izmir Ataturk Training and Research Hospital, a tertiary referral center in Turkey, were screened for eligibility for inclusion in this study.

Surgical management was individualized according to the anatomical extent of infection, neurological status, and stability requirements. The operative objectives generally included obtaining tissue samples for microbiological diagnosis, achieving source control through debridement and/or abscess drainage, performing neural decompression when indicated, and restoring or maintaining spinal stability with instrumentation in the presence of mechanical instability, deformity, or extensive osseous destruction.

### 2.1. Study Population

Adult patients (≥18 years) who underwent surgical intervention for a diagnosis of primary spinal infection and who had a minimum postoperative follow-up duration of at least 12 months were eligible for inclusion in this study. Primary spinal infection was defined as vertebral osteomyelitis, discitis/spondylodiscitis, and spinal canal infections (epidural, subdural, or intramedullary abscesses) confirmed by clinical, radiological, and microbiological findings. For the purposes of this study, “primary” refers to non-postoperative, non-implant-related infection at presentation; patients with postoperative surgical site infections and implant-related infections in the index segment were excluded due to the design of this study (patients who had undergone prior surgery in the same spinal segment were not eligible for inclusion). We did not routinely classify infections as community-acquired versus healthcare-associated. Patients were excluded if they met any of the following criteria (Supplementary Table S1):

- Had parasitic spinal infections (e.g., echinococcosis);
- Had a history of prior surgery in the same spinal segment;
- Had incomplete clinical data;
- Were lost to follow-up before 12 months.

### 2.2. Data Collection

Clinical data were retrospectively extracted from hospital electronic medical records and surgical archives. The following variables were recorded as candidate explanatory variables:

- Demographic Data: Age, sex, residence (urban vs. rural), and smoking status.
- Comorbidities: Diabetes mellitus, hypertension, cardiovascular disease, chronic kidney disease, chronic lung disease, rheumatologic disease, liver disease, malignancy, neuropsychiatric disorders, and other chronic systemic illnesses.
- Laboratory Parameters: Preoperative white blood cell count (WBC), platelet count (PLT), and serum albumin levels.
- Microbiological Data: Results of intraoperative tissue cultures and blood cultures.
- Perioperative Variables: Need for postoperative blood transfusion, steroid use, and length of hospital stay.
- Outcomes: In-hospital mortality; unplanned readmission (primary reason categorized as infection-related versus surgery-related/mechanical); and reoperation.

Per institutional practice, the management of primary spinal infection is multidisciplinary. Antimicrobial therapy was prescribed in consultation with the Infectious Diseases service and was tailored according to blood and intraoperative culture results and clinical response. Because of the retrospective design of this study and use of heterogeneous documentation, detailed infection-treatment process variables (e.g., specific agents, total duration of intravenous/oral therapy, outpatient adherence, use of local antibiotic adjuncts, standardized wound complication grading, and the extent/completeness of debridement or residual extra-spinal collections) were not consistently available and were therefore not analyzed in this study.

### 2.3. Outcome Definitions (Dependent Variables)

The primary outcome of interest was unplanned hospital readmission, defined as an unplanned inpatient readmission related to the index spinal infection episode or its surgical management, occurring within the following timeframes:

- Thirty days;
- Ninety days;
- One year after discharge following index surgery.

These endpoints were evaluated cumulatively at 30 days, 90 days, and 1 year after discharge. For the descriptive assessment of timing, readmissions were also grouped into 0–30 days, 31–90 days, and >90 days (91–365 days) after discharge.

Readmissions were further categorized as follows:

- Surgical Readmissions: Requiring reoperation. Reoperation was defined as any unplanned return to the operating room after discharge following the index surgery, regardless of whether antimicrobial therapy had been completed, and performed for infection recurrence/persistence in the index spinal segment or for surgery-related/mechanical complications (e.g., instrumentation failure). Planned staged or “second-look” procedures during the index hospitalization were not classified as readmissions or reoperations.
- Medical Readmissions: Managed without reoperation (i.e., non-operative inpatient care), primarily involving intravenous antibiotic therapy with close clinical and laboratory monitoring.

### 2.4. Statistical Analysis

All statistical analyses were performed using IBM SPSS Statistics Version 30.0 (IBM Corp., Armonk, NY, USA). Normally distributed continuous variables are expressed as mean ± standard deviation (SD), and non-normally distributed variables are expressed as median (interquartile range). Categorical variables are presented as frequencies and percentages. Univariate associations between candidate variables and hospital readmission were evaluated using the Chi-square test or Fisher’s exact test for categorical variables and Student’s *t*-test or the Mann–Whitney U test for continuous variables, as appropriate. For descriptive purposes, laboratory variables were also dichotomized using commonly applied clinical thresholds (WBC > 10,000/µL; PLT > 400,000/µL; serum albumin < 3.5 g/dL).

Given the limited number of readmission events observed in the cohort, multivariable logistic regression modeling was not performed to avoid model overfitting and unstable effect size estimation. Accordingly, this study was conducted as an exploratory, hypothesis-generating analysis that aimed to describe readmission patterns and identify potential univariate associations rather than establish causal or predictive inference. Alternative approaches for rare-event outcomes (e.g., penalized regression or Firth’s bias-reduced logistic regression) can be considered to reduce small-sample bias and separation [11,12]; however, with only seven readmission events and multiple candidate covariates, adjusted models would remain highly unstable and model-dependent. The limited event number also reduces statistical power and increases the risk of type II error (false-negative associations); therefore, non-significant findings should be interpreted with caution.

A *p*-value of <0.05 was considered statistically significant.

### 2.5. Ethics Approval

This study was approved by the Izmir Katip Celebi University Health Research Institutional Review Board (approval number: 0357, date: 16 January 2025). Due to the retrospective nature of the study and use of anonymized data, the requirement for informed consent was waived.

## 3. Results

### 3.1. Baseline Patient Characteristics

A total of 79 adult patients (49 males and 30 females) who underwent surgical treatment for primary spinal infection were included in this study. The mean age of the patients was 62.2 ± 12.7 years. Most patients resided in urban areas (64.6%), and 17.7% were active smokers. At least one chronic systemic disease was present in 65.8% of the cohort. The demographic characteristics of the patients are shown in Table 1.

### 3.2. In-Hospital Mortality

Four patients (5.1%) died during the index hospitalization. Among these patients, *Staphylococcus aureus* was isolated in one case, whereas no microbial growth was detected in the remaining three. A total of 75 patients survived the initial hospitalization and were discharged.

### 3.3. Readmission and Reoperation Outcomes

At 1-year follow-up, seven patients (10.3%) experienced an unplanned hospital readmission related to the index spinal infection episode. All readmissions involved the index spinal segment and were attributable to infection recurrence/persistence (n = 6) or instrumentation-related mechanical failure (n = 1); no readmissions for unrelated medical diagnoses were observed. Among these patients, four (5.9%) required reoperation, while three were managed medically without reoperation, primarily with inpatient intravenous antibiotic therapy and close monitoring.

### 3.4. Early Period (≤30 Days)

Five patients were readmitted within the first 30 days after discharge. Among these patients, three required reoperation due to recurrent spinal infection, whereas two were managed medically without reoperation using intravenous antibiotic therapy.

### 3.5. Intermediate Period (31–90 Days)

No hospital readmissions occurred between postoperative days 31 and 90. Accordingly, the cumulative 90-day readmission rate in this cohort was identical to the 30-day readmission rate.

### 3.6. Late Period (>90 Days)

Two additional patients were readmitted after 90 days. One patient required reoperation due to instrumentation failure (rod fracture), and one patient was readmitted for infection-related intravenous antibiotic therapy without surgical intervention.

### 3.7. Microbiological Findings

Among the patients who underwent reoperation in the early period, intraoperative cultures revealed the following findings:

- *Staphylococcus aureus* was present in one patient;
- *Klebsiella pneumoniae* was present in one patient;
- *Mycobacterium tuberculosis* was present in one patient.

In patients who did not require readmission within the first year, microbiological cultures demonstrated a broad pathogen spectrum. The most frequently isolated organism was *Staphylococcus aureus* (n = 8), followed by *Staphylococcus epidermidis* (n = 4) and *Mycobacterium tuberculosis* (n = 5). Single cases *of Pseudomonas aeruginosa*, *Bacillus* spp., *Corynebacterium* spp., *Escherichia coli*, *Klebsiella pneumoniae*, *Streptococcus suis*, *Staphylococcus kloosii*, *Streptococcus mitis*, *Serratia marcescens*, and *Micrococcus luteus* were also identified.

### 3.8. Exploratory Univariate Association Analysis

According to the findings of the exploratory univariate analyses, none of the evaluated demographic, clinical, laboratory, perioperative, or microbiological variables were significantly associated with unplanned readmission at 30 days, 90 days, or 1-year follow-up (all *p* > 0.05).

## 4. Discussion

In this study, we evaluated a comprehensive set of demographic, clinical, laboratory, perioperative, and microbiological variables as potential predictors of early and late unplanned hospital readmission following surgical treatment of primary spinal infections. The principal finding of our exploratory analysis is that none of the variables we evaluated demonstrated a statistically significant association with readmission in univariate comparisons at 30-day, 90-day, or 1-year follow-up. This negative result highlights the multifactorial and heterogeneous nature of postoperative failure and readmission in patients with PSIs and underscores the limitations of relying on isolated clinical parameters for risk stratification in this complex disease entity.

Notably, all readmissions within the first 90 days occurred within the first 30 days after discharge, with no events observed in the 31–90-day interval; therefore, the cumulative 90-day readmission rate mirrored the 30-day rate in this cohort. We retained the 90-day endpoint because it is a commonly reported surgical quality metric, which allowed for the findings of this study to be compared with those of prior studies; however, in the present cohort, it did not provide additional discriminatory information beyond the 30-day analysis. The absence of intermediate-period events may reflect the limited event number and the possibility that clinically relevant failures tended to manifest either early (≤30 days) or later (>90 days).

Hospital readmission following spine infection surgery reflects a convergence of multiple biological, mechanical, and systemic factors rather than a single dominant driver. Unlike elective degenerative spine surgery, patients with PSIs frequently present with impaired host immunity, persistent inflammatory burden, and ongoing systemic vulnerability. Prior studies evaluating readmission after general spine surgery have identified age, nutritional status, renal dysfunction, and comorbidity burden as potential predictors; however, PSI-specific data remain sparse and inconsistent. Recent national database studies focused on spondylodiscitis have reported high 90-day all-cause readmission rates (~35%) and suggest that index-admission surgical management may reduce readmission risk, while comorbidities such as diabetes may increase risk [9,10]. Our findings suggest that risk prediction models derived from mixed spine surgery cohorts may not be directly applicable to surgically treated PSI populations. In a study evaluating postoperative readmissions after general surgery, older age was associated with increased risk in some subgroups, but this was not an independent predictor across all procedures [13].

Hypoalbuminemia is widely accepted as a surrogate marker of poor nutritional reserve and systemic inflammation and has been associated with increased postoperative morbidity, infection, and readmission in multiple surgical disciplines [14]. In the present cohort, although patients with low preoperative albumin levels demonstrated numerically higher readmission rates, this association did not reach statistical significance. Similarly, the high platelet count group (above 400,000/µL) did not differ significantly from the low platelet count group (400,000/µL or lower) in terms of readmission rates (*p* = 0.490). In the literature, elevated inflammatory markers, including WBC, have been associated with higher postoperative risk [15]; however, we did not detect a relationship between elevated WBC count (above 10,000/µL) and the readmission rate (*p* = 0.167). These findings support the notion that static preoperative laboratory variables may inadequately reflect the complex and evolving biological environment of spinal infection.

Geographic and socioeconomic factors, including urban residence and access to care, have been associated with readmission risk [16]. In terms of demographic characteristics, gender, place of residence (urban vs. rural), and smoking status were not significantly associated with readmission.

Diabetes and other comorbidities have been shown to increase the risk of surgical complications and unplanned readmissions [17]. When comorbid conditions were assessed, common morbidities such as chronic lung disease, cardiovascular disorders, diabetes, hypertension, and renal failure were not significantly associated with readmission rates. For example, the readmission rate among patients with diabetes was 5.1% compared to 3.8% among those without diabetes, but this difference was not statistically significant (*p* = 0.561). These findings suggest that comorbidities like diabetes could be potential risk factors, but the limited sample size within this study might have prevented statistical significance being reached.

Perioperative and postoperative steroid use and blood transfusions were also analyzed. Blood transfusions are associated with an increased risk of infection and readmission [18]. Steroid use can also impair wound healing and predispose patients to infections [19]. In this study, both variables were more frequent among readmitted patients, but differences were not statistically discernible. These results may reflect limited statistical power rather than the true absence of an effect.

The microbiological spectrum observed in this cohort was highly heterogeneous, ranging from common Gram-positive pathogens such as *Staphylococcus aureus* and *Staphylococcus epidermidis* to Gram-negative and mycobacterial organisms. Patients who underwent reoperation demonstrated a concentration of high-virulence or difficult-to-eradicate pathogens, including *Staphylococcus aureus*, *Klebsiella pneumoniae*, and *Mycobacterium tuberculosis*. This observation reinforces the concept that treatment failure in PSI is often organism-driven, particularly in the presence of multidrug resistance or atypical pathogens [20,21]. These findings underscore the critical importance of timely microbiological diagnostics, resistance profiling, and individualized antimicrobial stewardship programs [22,23]. The absence of microbial growth in several fatal cases further highlights the diagnostic and therapeutic challenges associated with culture-negative spinal infections.

Several factors likely contributed to these null findings. Firstly, the low absolute event number limits statistical power for detecting modest effect sizes. Secondly, PSI represents a biologically heterogeneous disease spectrum, encompassing diverse etiologies (e.g., pyogenic versus tuberculous), anatomical levels and compartments (cervical, thoracic, lumbar; vertebral body/disk space, epidural/paraspinal), microbiological profiles, and surgical strategies (including varying use of instrumentation). Without stratification or adjustment for these sources of variability, subgroup-specific effects may be diluted, limiting the ability to detect meaningful associations. Thirdly, the mechanisms underlying readmission often involve dynamic, time-dependent interactions between infection control, mechanical stability, and systemic recovery, which cannot be fully captured by baseline variables alone. Collectively, these features suggest that traditional univariable risk factor analysis may be insufficient to model readmission risk in PSI populations.

In addition, reliance on univariate comparisons limits interpretability because potential confounding cannot be addressed, and the evaluation of multiple candidate variables increases the likelihood of chance findings. Conversely, true associations may remain undetected due to limited power. The commonly cited “10 events per variable” heuristic is conservative and may be relaxed for parsimonious models [24]. For rare-event binary outcomes, bias-reduced and penalized approaches (such as Firth’s logistic regression or other penalized likelihood/regularized regression methods) may yield more stable estimates than standard maximum-likelihood logistic regression, particularly in the presence of separation [11,12]. Nevertheless, even these methods require careful prespecification of a small set of clinically prioritized covariates and adequate event information; in our cohort, the very low event number would likely produce model-dependent estimates with wide uncertainty. Accordingly, we framed our analyses as exploratory and emphasize that future multicenter studies with larger event counts should consider these approaches to enable adjusted inference.

Moreover, variation in surgical approach and operative objectives may substantially influence postoperative infection control and the likelihood of readmission. In primary spinal infection, index surgery may range from diagnostic biopsy and abscess drainage to extensive debridement, neural decompression, and instrumented stabilization aiming to restore mechanical integrity [8,22,25,26]. Because operative strategies were tailored to patient- and disease-specific anatomy and the number of readmission events was limited, we did not perform stratified subgroup analyses by surgical approach or operative objective; it is important that these are performed in larger multicenter cohorts. Notably, similar null findings have been reported even in postoperative spinal surgical site infection cohorts that incorporated surgical and antibiotic-related variables and applied multivariable adjustment. For instance, in their study, Billières et al. found that none of their variables were associated with remission in cluster-controlled multivariate Cox regression, including infection due to *Staphylococcus aureus*, the number of surgical debridements, local antibiotic therapy, and total antibiotic duration [27].

In addition to baseline host factors, infection-treatment process variables may be associated with recurrence and subsequent readmission in PSIs. These include the adequacy of source control (e.g., completeness of debridement and drainage and the presence of residual paraspinal or extra-spinal collections such as psoas abscesses), postoperative wound breakdown or surgical site infection after the index operation, pathogen virulence and resistance profiles, timing of active antimicrobial therapy, total treatment duration and route transitions, and adherence to prolonged therapy. Another clinically relevant distinction is relapse (same pathogen in the same anatomical segment) versus reinfection due to a new organism or a remote infectious source (e.g., urinary tract infection or catheter-related bacteremia). Because these parameters were not available in a standardized manner in this retrospective dataset—particularly for medical readmissions without repeat surgery—we could not evaluate their association with the outcomes. Future prospective studies should aim to systematically capture these infection-specific variables alongside surgical factors to better explain treatment failure and readmission.

### 4.1. Implications for Clinical Practice and Future Risk Modeling

Our findings highlight the potential value of multimodal and dynamic risk modeling approaches, potentially incorporating longitudinal laboratory trends, advanced imaging parameters, pathogen resistance profiles, and perioperative physiological trajectories. Artificial intelligence-based predictive systems and multicenter datasets with greater statistical power may be essential to better characterize correlates of readmission in PSI surgery. Until such models are developed, clinicians should maintain a high index of suspicion for readmission across all PSI patients rather than relying on isolated preoperative risk factors.

From a clinical standpoint, the absence of statistically significant associations in our exploratory analyses should not be interpreted as an absence of risk; rather, it indicates that we could not identify a robust low-risk subgroup that would justify reduced surveillance. Therefore, a universal and structured postoperative follow-up strategy for all surgically treated PSI patients may be preferable to selective risk-based surveillance. Given that most readmissions in our cohort occurred within 30 days, early post-discharge contact and timely outpatient review during ongoing antimicrobial therapy may facilitate the earlier recognition of recurrence or complications. Clinicians may also consider maintaining a low threshold for repeat laboratory assessment and imaging when symptoms recur or when clinical/laboratory trajectories fail to improve, with close coordination between spine surgeons and infectious disease teams, optimization of comorbidities and nutritional status, and monitoring for instrumentation-related complications when applicable [22,23].

### 4.2. Limitations

This study has several important limitations that should be acknowledged. Firstly, its retrospective, single-center design inherently introduces selection bias and limits the generalizability of the findings. Secondly, although the overall cohort size is acceptable for an exploratory analysis, the absolute number of readmission events was relatively low (n = 7 within 1 year), which restricts statistical power, increases the risk of type II error, and precludes reliable multivariable risk modeling. Thirdly, we did not adjust for potential confounders or perform time-to-event modeling. Although the traditional “10 events per variable” heuristic is conservative and may be relaxed for parsimonious models [24], the combination of very low event information and the incomplete capture of key infection-treatment covariates in our cohort would still likely yield model-dependent estimates with wide uncertainty, even with bias-reduced or penalized approaches (e.g., Firth’s logistic regression). Fourthly, PSI is clinically heterogeneous; however, we were unable to stratify or adjust for several important sources of variability (e.g., infection etiology such as pyogenic versus tuberculous disease, anatomical level/extent and compartment involvement, and the use of instrumentation), and we did not perform subgroup analyses according to surgical approach or operative objectives. The limited event number prevented the performance of meaningful subgroup analysis.

Additionally, readmissions were identified from institutional records; readmissions managed at external hospitals (or post-discharge emergency visits not resulting in admission) may have been missed, potentially underestimating the true event rate. Several potentially relevant clinical variables were not available in a standardized form (e.g., detailed radiological severity/extent, abscess size, and inflammatory markers such as C-reactive protein or erythrocyte sedimentation rate), limiting the comprehensiveness of the assessment of potential associations. Finally, we did not perform a detailed evaluation of antimicrobial regimens (agent selection, duration, outpatient parenteral antibiotic therapy, or adherence), which may be associated with recurrence and readmission. Collectively, these limitations support the need for larger prospective multicenter studies with standardized data collection. We also did not systematically capture immediate postoperative wound complications or classify readmissions as surgical site infections attributable to the index operation. Surgeon- and procedure-level variables (e.g., operator grade/supervision, after-hours surgery), the number of debridement procedures per infection episode, and standardized measures of adequacy/completeness of debridement and residual extra-spinal collections were not available. In addition, we did not systematically differentiate relapse from reinfection or evaluate potential remote sources of new infection, nor did we capture intervening events during follow-up (e.g., trauma or ongoing substance use) that could have contributed to rehospitalization. These unmeasured infection- and treatment-related factors may contribute to outcome variability and further limit causal interpretation. We also did not systematically classify infections as community-acquired versus healthcare-associated/nosocomial, and information regarding history of pre-infection spinal procedures such as epidural steroid injections was not consistently available.

## 5. Conclusions

In conclusion, in this exploratory retrospective cohort, none of the demographic, clinical, laboratory, perioperative, or microbiological variables that we evaluated were significantly associated with unplanned hospital readmission following the surgical treatment of primary spinal infections in the univariate analyses. These findings underscore the multifactorial and dynamically evolving nature of postoperative failure and readmission in this complex patient population, though they should be interpreted as hypothesis-generating given the low number of readmission events. Until robust risk stratification models are available, universal close postoperative surveillance for surgically treated PSI patients should be considered rather than selective risk-based follow-up. The performance of prospective, multicenter studies with larger sample sizes and standardized follow-up protocols in the future is essential to validate these findings and develop evidence-based strategies that aim to minimize readmissions and improve the long-term outcomes for this high-risk population.

## Figures and Tables

**Table 1:** Baseline demographic and clinical characteristics of patients.

	n	%
Age (years), mean ± SD	62.2 ± 12.7	
Gender (male)	49	62
Region		
Urban	51	64.6
Rural	27	34.2
Undefined	1	1.3
Comorbidities		
Chronic lung disease	4	5.1
Rheumatologic disease	5	6.3
Chronic liver disease	2	2.5
Cardiovascular disease	26	32.9
Diabetes mellitus	33	41.8
Hypertension	33	41.8
Chronic kidney disease	9	11.4
Psychiatric disorder	3	3.8
Malignancy	3	3.8
Alzheimer’s disease	1	1.3
Osteogenesis imperfecta	1	1.3
≥1 chronic systemic disease	52	65.8
Active smoking	14	17.7
Platelet count (PLT)		
≤400,000/µL	65	82.2
>400,000/µL	14	17.8
Serum albumin		
<3.5 g/dL	50	63.3
>3.5 g/dL	29	36.7

## Data Availability

The data presented in this study are available from the corresponding author upon reasonable request in accordance with ethical and privacy restrictions.

## Supplementary Materials

The following supporting information can be downloaded at: https://www.mdpi.com/article/10.3390/jcm15041600/s1, Figure S1: This figure provides an illustrative case example of early postoperative recurrence and long-term outcomes following repeat surgical debridement and drainage. A 61-year-old male patient was discharged 22 days after undergoing his first surgery. However, he was readmitted 28 days after discharge and found to have a recurrence of the lumbar spinal abscess. The patient underwent reoperation, drainage, and debridement. Clinical and radiological follow-up 3 years after the second surgery revealed complete recovery and no recurrence. Tissue samples obtained from both surgeries grew methicillin-sensitive Staphylococcus aureus (MSSA). The patient’s sagittal T2-weighted MRI images from (a) before the first surgery, (b) before the second surgery, and (c) three years after the second surgery are shown; Table S1: Study eligibility criteria.

## Abbreviations

The following abbreviations are used in this manuscript:

PSI	Primary Spinal Infection
WBC	White Blood Cell
PLT	Platelet Count
MRI	Magnetic Resonance Imaging
MSSA	Methicillin-Sensitive *Staphylococcus aureus*
MRSA	Methicillin-Resistant *Staphylococcus aureus*
