# Supplementary material for: Exploratory Analysis of Factors Affecting 30-Day, 90-Day, and 1-Year Readmission After Surgical Treatment of Primary Spinal Infection in Adults
Source: J Clin Med. 2026 Feb 19;15(4):1600. doi: 10.3390/jcm15041600 (PMC12942489; doi:10.3390/jcm15041600)
Supplement: Supplementary file 1 [file jcm-15-01600-s001.zip › jcm-4105047-supplementary.pdf]

Supplementary Materials

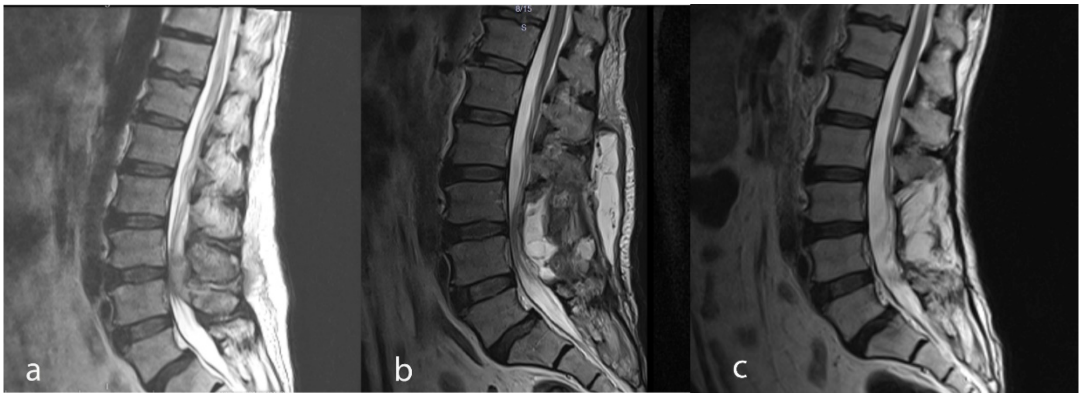

Figure S1: This figure provides an illustrative case ex-ample of early postoperative recurrence and long-term outcome following repeat surgical debridement and drainage. A 61-year-old male patient was discharged 22 days after his first surgery. However, he was readmitted 28 days after discharge and was found to have a recurrence of the lumbar spinal abscess. The patient underwent reoperation, drainage, and debridement. Clinical and radiological follow-up 3 years after the second surgery revealed complete recovery and no recurrence. Tissue samples obtained from both surgeries grew *methicillin-sensitive Staphylococcus aureus* (MSSA). The patient's sagittal T2-weighted MRI images are shown. **(a)** Before the first surgery, **(b)** Before the second surgery, **(c)** Three years after the second surgery.

Supplementary Table S1. Study eligibility criteria

Inclusion criteria	Exclusion criteria
Adults (≥18 years)	Parasitic spinal infection (e.g., echinococcosis)
Surgical treatment for primary spinal infection (PSI)	Prior surgery in the same spinal segment
Index surgery performed between January 2017 and December 2023	Incomplete clinical data
Minimum postoperative follow-up ≥12 months	Lost to follow-up before 12 months
Primary PSI at presentation (non-postoperative, non–implant-related)	Postoperative or implant-related infection at presentation
